# Atherosclerosis Plaque Reduction by Lycopene Is Mediated by Increased Energy Expenditure through AMPK and PPARα in ApoE KO Mice Fed with a High Fat Diet

**DOI:** 10.3390/biom12070973

**Published:** 2022-07-12

**Authors:** Federica Mannino, Giovanni Pallio, Domenica Altavilla, Francesco Squadrito, Giovanna Vermiglio, Alessandra Bitto, Natasha Irrera

**Affiliations:** 1 Department of Clinical and Experimental Medicine, University of Messina, Via C. Valeria, 98125 Messina, Italy; fmannino@unime.it (F.M.); gpallio@unime.it (G.P.); fsquadrito@unime.it (F.S.); nirrera@unime.it (N.I.); 2 Department of Biomedical, Dental, Morphological and Functional Imaging Sciences, University of Messina, Via C. Valeria, 98125 Messina, Italy; daltavilla@unime.it (D.A.); giovanna.vermiglio1@unime.it (G.V.); 3 SunNutraPharma s.r.l., Spin-Off Company of University of Messina, Via C. Valeria, 98125 Messina, Italy

**Keywords:** atherosclerosis, lycopene, nutraceuticals, oxidative stress, PPARα

## Abstract

Lycopene is a carotenoid found in tomatoes that has potent antioxidant activity. The Mediterranean diet is particularly rich in lycopene, which has well-known beneficial effects on cardiovascular health. We tested the effects of lycopene extract in a group of 20 ApoE knockout mice, fed with a high fat western diet for 14 weeks. Starting from week 3 and up to week 14, the mice were randomly divided into two groups that received lycopene (*n* = 10) by oral suspension every day at the human equivalent dose of 60 mg/day (0.246 mg/mouse/day), or the vehicle solution (*n* = 10). The lycopene administration reduced triglycerides and cholesterol blood levels starting from week 6 and continuing through to the end of the experiment (*p* < 0.001). This reduction was mediated by an enhanced liver expression of PPAR-α and AMPK-α and reduced SREBP levels (*p* < 0.0001). As a histological red-out, the extent of atherosclerotic plaques and the intima–media thickness in the aorta were significantly reduced by lycopene. In this context, lycopene augmented the Nrf-2 positivity staining in the endothelium, thereby confirming that its antioxidant activity was mediated by this nuclear factor. The positive results obtained in this pre-clinical model further support the use of lycopene extracts to reduce atherosclerosis.

## 1. Introduction

Atherosclerosis is a chronic inflammatory disease that is responsible for several cardiovascular events such as ischemic heart disease, coronary artery disease, peripheral artery disease, and ischemic stroke [1]. The prevalence of atherosclerosis is increasing worldwide and due to the aging population the incidence of cardiovascular diseases is expected to rise, together with disease-related deaths and disabilities. A male gender, smoking, and alcohol abuse, together with a high fat diet (HFD), are considered the factors associated with an increase in the prevalence rate from 1.655 per 100,000 inhabitants to 1.845/100,000, or more by 2030 [2]. The atherosclerotic process is characterized by lipid deposition accompanied with smooth muscle cells and fibrous matrix proliferation, which gradually develop into the formation of an atherosclerotic plaque that causes vascular stenosis, and when unstable, may fragment and cause thrombosis or embolism [3,4,5].

There is a large body of evidence supporting the role of AMPK (adenosine monophosphate-activated protein kinase) as an important factor for attenuating atherosclerosis development. This kinase was commonly known as a positive regulator of fatty acid oxidation that regulates multiple physiological processes including lipid and glucose metabolism and the normalization of energy imbalances [6,7].

A previous paper showed that the ability of polyphenols to stimulate AMPK and reduce hepatocellular lipid accumulation could be important for reducing atherosclerosis, at least in experimental models [7]. Among the natural activators of AMPK, lycopene has emerged in the past decades since it has been proven to modulate oxidative stress and the cytokine production involved in chronic processes such as cardiovascular diseases [8]. The protective beneficial effects of lycopene have been mainly attributed to its capacity to prevent atherogenesis through its antioxidant properties [9,10].

Additionally, lycopene decreased cholesterol absorption in the intestine and prevented atherosclerosis progression in a well-studied atherosclerosis model, which involved apolipoprotein E knockout (ApoE^−/−^) mice fed with HFD [11]. Moreover, lycopene strongly reduced cholesterol and triglyceride serum levels in ApoE*3Leiden mice fed with an atherogenic diet [12]. Lycopene’s beneficial effects, which have been achieved in studies on several cell types and under multiple experimental conditions, are widely known and summarized in previous papers, supporting the importance of this carotenoid for metabolic derangements and cardiovascular diseases [13,14]. In addition, in human studies, patients using lycopene supplementation demonstrated an increased metabolic profile in terms of reduced plasma low-density lipoprotein (LDL) and cholesterol levels [15,16].

As further a mechanism of action that could be useful in atherosclerosis development, lycopene can regulate hepatic lipid metabolism through SIRT-1 (sirtuin-1) activation, thus positively regulating the transcriptional activity of both the peroxisome proliferator-activated receptor alpha (PPARα) and the peroxisome proliferator-activated receptor gamma coactivator 1-alpha (PGC-1α), which are involved in fatty acid catabolism [17]. The involvement of the SIRT1/PPARα/PGC-1α axis in lycopene’s mode of action, with a specific focus on hepatic steatosis and glucose metabolism, was confirmed in mice that over-expressed SIRT-1 and were fed with HFD [18]. SIRT1 may also regulate hepatic fatty acid utilization by reducing the expression of the transcription factor sterol regulatory element–binding proteins (SREBP-1), a positive regulator of fatty acid synthesis, and promoting the phosphorylation/activation of AMPK that regulates fatty acid oxidation, thus preventing lipid accumulation [7].

The effects of lycopene on this specific pathway in the context of atherosclerosis are not yet fully described; therefore, the aim of this study was to analyze the effects of a lycopene extract from Sicilian cherry tomatoes on atherosclerosis development in ApoE KO mice fed with HFD.

## 2. Material and Methods

### 2.1. Animals and Drugs

All animal procedures were in accordance with the Principles of Laboratory Animal Care (NIH publication no.85-23, revised 1985), authorized by the Ministry of Health Review Board for the care of laboratory animals (approval number 63/2017), and were in accordance with the ARRIVE Guidelines [19]. A total of 30 male ApoE^−/−^ mice 12 weeks old (strain B6.129P2-Apoe^tm1Unc^/J) were purchased from Charles River Laboratories (Calco, Milan, Italy). Animals were maintained in the animal facility of the Department of Clinical and Experimental Medicine of the University of Messina in standard cages and environmental conditions with water and food ad libitum. After 1 week of acclimation, animals were randomly assigned to 3 groups of 10 animals each; 1 group was fed with a normal diet for 14 weeks (control group) and the other 2 groups received high fat diet food (SAFE U8958 Version 35; Safe, Rosenberg, Germany) and were orally administered (in a fluid suspension given with a plastic pipette) with lycopene (0.264 mg/mouse/day) or vehicle, starting from week 3 and up to week 14.

Lycopene was obtained with 90% of purity by the cold-extraction method from Sicilian cherry tomatoes (that have the highest content in lycopene ≈90 μg/g) and was prepared fresh daily, dissolved in corn oil, mixed with 1% sucrose to improve its palatability (thus, mice were licking the fluid suspension from the plastic pipette), and administered every 24 h. The dose was chosen according to a pilot study that demonstrated the efficacy of a dietary supplementation (60 mg/day) in reducing plasma cholesterol in healthy young men [20] and was calculated according to the following formula AED (mg/kg) = Human does (mg/kg) × Km ratio.

Daily measurements included body weight, food, and water intake; in addition, at selected time points, a couple of drops of blood were obtained from the jugular vein and immediately used for lipid measurement. At the end of the experiment, the animals were anesthetized with a supramaximal dose of ketamine (100 mg/kg) and xylazine (10 mg/kg) and killed by exsanguination, and the blood taken from the heart was immediately used for lipid measurement. Following saline perfusion, liver and aortic samples were removed and stored for histological assessments and biochemical marker measurement.

### 2.2. Measurement of Cholesterol and Triglyceride Levels

The concentrations of total cholesterol and the triglyceride blood content were measured at 0, 2, 4, 6, and 14 weeks using the MultiCare-IN kit (Biochemical System Intern. Arezzo, Italy). Cholesterol detection range: 130–400 mg/dL; triglyceride detection range: 50–500 mg/dL.

### 2.3. Quantification of Atherosclerosis

At the end of the experiment, mouse aortas were harvested for atherosclerosis quantification following the American Heart Association’s statement on atherosclerosis. Aortas were perfused with saline by left ventricular puncture and were fixed in 10% formalin overnight. Adventitial fat was removed, and atherosclerosis was quantified on intima on the entire aorta (the arch, the thoracic, and the abdominal sections) using Image J software to calculate the percentage of atherosclerotic plaque surface area [21].

### 2.4. Histological Evaluation of Aortas

After exsanguination, the hearts with 1–1.5 cm of ascending thoracic aorta were removed from the animals and fixed in 10% buffered formalin, processed for paraffin embedding, sectioned at 5 μm, and subsequently stained with hematoxylin and eosin for examination under a light microscope (Leica microsystem, Wetzlar, Germany). Aortic intima–media thickness was measured from the endothelial surface to the adventitia in 10 different fields for each sample, using the Leica image software. The histological score was based on the microscopic appearance of the aortas considering (i) the loss of endothelial integrity, (ii) the presence of inflammatory infiltrate, (iii) the infiltration of the sub-intimal layer, (iv) the presence of red blood cells and fibrin deposition in the plaque, and (v) the presence of a clot in the sub-intimal area. Each of the above parameters were scored as follows: absent 0, mild 0.5, moderate 1, and severe 1.5, with a total range between 0 and 7.5.

### 2.5. Immunofluorescence Evaluation of Nrf2

Sections of aorta were rehydrated and incubated in sodium citrate buffer (10 mM Sodium citrate; 0.05% Tween 20; pH 6.0) in a microwave at 750 W for antigen retrieval. Once rehydrated, slides were pre-incubated with 0.3% Triton X-100 in PBS for 10 min and in PBS/albumin (1%) for 20 min. Primary antibodies for Nrf-2 and CD34 (1:150 and 1:100 dilution, respectively; Abcam, Cambridge, UK) were used to evaluate possible co-localization in endothelial cells. After washing three times with PBS for 10 min, Texas Red and Fitc-conjugated IgG (1:100 dilution; Jackson ImmunoResearch Laboratories, West Grove, PA, USA) were used for Nrf-2 and CD34 detection, respectively. Sections were sealed with mounting medium following incubation with DAPI (1:1000 dilution; Sigma Chemicals, St. Louis, MO, USA) for 10 min in the dark at RT to label nuclei. Images were collected from a Zeiss LSM 510 confocal microscope equipped with Argon laser (458 and 488 nm λ) and two HeNe laser (543 and 633 nm λ) as previously described [22].

### 2.6. Real-Time Quantitative PCR Amplification (RTqPCR)

Total RNA was extracted from the liver tissues at the end of the experimental procedures using Trizol LS reagent (Invitrogen, Carlsbad, CA, USA). Total RNA (1 μg) was reverse transcribed in a final volume of 20 μL using a Superscript VILO kit (Invitrogen). The cDNA (1 μL) was added to the EvaGreen qPCR Master Mix (Biotium Inc., Fremont, CA, USA) at a final volume of 20 μL per well. Samples were run in duplicate, and β-actin was used as the housekeeping gene; the reaction was performed using the 2-step thermal protocol recommended by the manufacturer. The final primer concentration selected to perform the analysis was 10 μM. Target genes were PPARα, SREBF-1, and AMPK-α. Results were calculated using the 2^−^^ΔΔCT^ method and expressed as n-fold increase in gene expression using the control group as calibrator [23,24,25]. Primers used for targets and reference genes are listed in Table 1.

### 2.7. Isolation of Total Proteins and Western Blot Analysis

Protein extraction was performed in liver for Western Blot analysis, as previously described [26,27]. About 30 μg of proteins were loaded and specific antibodies were used to evaluate PPARα (ab61182), SREBP-1 (ab28481) (1:250 and 1:1000 dilution respectively; Abcam, Cambridge, MA, USA), p-AMPK-α (2535), and β-actin (4970) (1:1000 dilution each; Cell Signaling, Beverly, MA, USA) which was used as loading control. The images were obtained using specific software (C-DiGit Blot Scanner with Image Studio 4.0 software, LI-Cor, Lincoln, NE, USA), and densitometric data were expressed as integrated intensity, following evaluation of a region of interest (ROI) identical for all samples within the same blot.

### 2.8. Statistical Analysis

All quantitative data are expressed as mean ± SD for each group. Longitudinal data were compared by two-way ANOVA with Sidak post-test for intergroup comparisons. A standard Student’s *t*-test was used for western blot data. One-way ANOVA was used to compare q-PCR and % lesion area data. Histological non-parametric data were analyzed using the Mann–Whitney U test. Statistical significance was set at *p* < 0.05. Graphs were drawn using GraphPad Prism software version 8.0 for macOS (GraphPad Software Inc., La Jolla, CA, USA).

## 3. Results

### 3.1. Lycopene Effects on Body Weight and Food Intake

The body weights and food intake were recorded at baseline and throughout the experimental period. All animals showed a physiological increase in body weight during the treatment period (from 25 ± 4 to 30 ± 5 g); additionally, considering food intake normalized over the weights (5 ± 1.5 g/mouse/day), no differences were observed among the three experimental groups.

### 3.2. Lycopene Effects on Triglycerides and Cholesterol Levels

**Figure 1:**
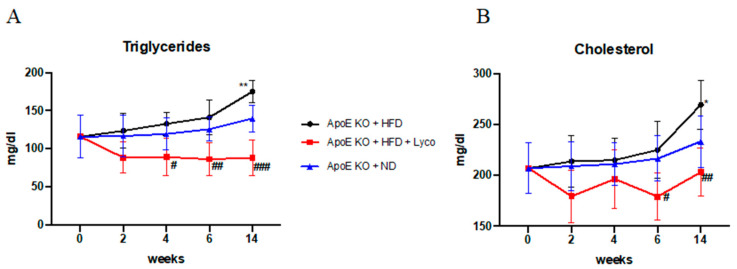
Triglyceride and cholesterol levels in blood evaluated during the experimental period. Values were obtained from 10 animals per group and are expressed as mean ± SD. Triglycerides (**A**) ** *p* < 0.001 vs. ApoE KO + ND; # *p* < 0.05 vs. ApoE KO + HFD; ## *p* < 0.001 vs. ApoE KO + HFD; ### *p* < 0.0001 vs. ApoE KO + HFD. Cholesterol (**B**) * *p* < 0.05 vs. ApoE KO + ND; # *p* < 0.05 vs. ApoE KO + HFD; ## *p* < 0.001 vs. ApoE KO + HFD.

Male ApoE^−/−^ mice were fed with a normal diet or HFD for 14 weeks; the HFD animals were treated daily with either the vehicle or lycopene starting from week 3 and up to week 14. The HFD group showed a significant increase in both triglycerides and cholesterol levels at week 14 compared to the normal diet group. The lycopene treatment significantly reduced the triglyceride levels in the blood of the mice fed with HFD from week 4 (*p* < 0.05 vs. HFD; Figure 1A), showing a decrease in triglycerides during all the experimental periods (*p* < 0.0001 at the end of experiment vs. HFD; Figure 1A). Moreover, the lycopene administration significantly reduced cholesterol levels in the blood from week 6 to the end of the experiment (*p* < 0.05 at week 6 vs. HFD; *p* < 0.001 at week 14 vs. HFD; Figure 1B).

### 3.3. Effects of Lycopene Treatment on Atherosclerotic Lesions

**Figure 2:**
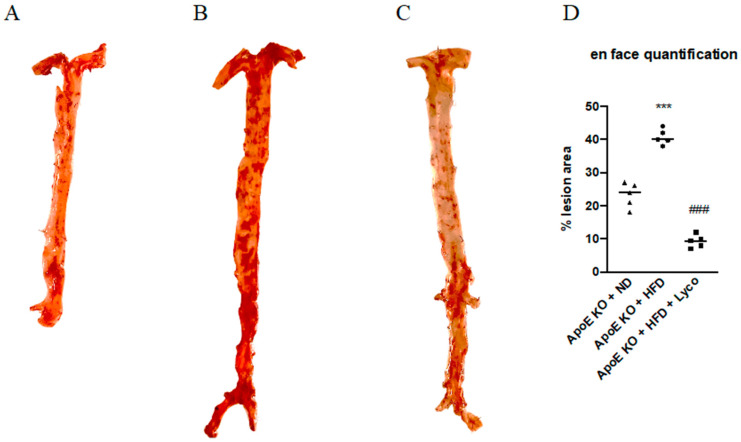
Effects of Lycopene on percentage of lesion surface area in aortas samples stained with oil-red-o from ApoE KO + ND (**A**), ApoE KO + HFD (**B**), and ApoE KO + HFD + Lyco (**C**) groups. The graph in (**D**) shows the quantification of the percentage of atherosclerotic plaque surface area. Values were obtained from 5 animals per group and are expressed as the means ± SD. *** *p* < 0.0001 vs. ApoE KO + ND; ### *p* < 0.0001 vs. ApoE KO + HFD.

The atherosclerotic plaques were quantified by Oil Red O staining of the intimal surfaces of the entire aorta (the arch, the thoracic, and the abdominal sections of the ApoE animals). The results showed a difference in the lesion areas between groups; in particular, the number of atherosclerotic lesions was significantly increased in the ApoE KO + HFD compared to the ApoE KO + ND group, whereas the ApoE KO + HFD + Lycopene group showed a marked reduction of atherosclerotic lesions compared to the ApoE KO + HFD group (Figure 2). The quantification of the percentage of atherosclerotic plaque surface area is summarized in Figure 2D.

### 3.4. Lycopene Reduced Histological Damage

**Figure 3:**
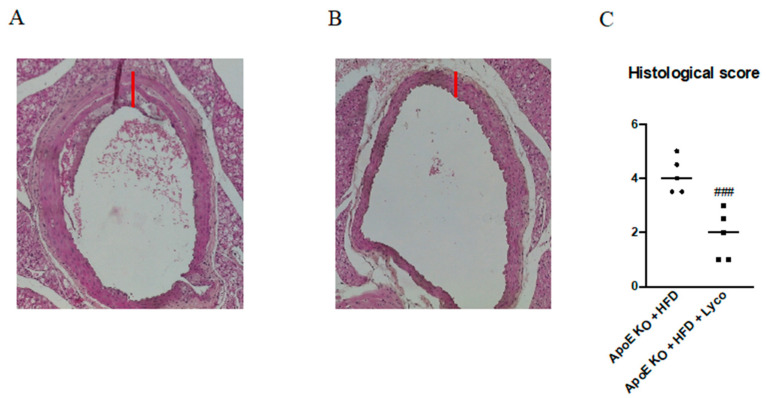
Representative H&E images (original magnification 10×) of thoracic aortas from ApoE KO + HFD (**A**) and ApoE KO + HFD + Lyco (**B**). The graph in (**C**) represents the histological score. Values are expressed as the mean ± SD of 5 animals. ### *p* < 0.0001 vs. ApoE KO + HFD.

To evaluate the histological characteristics of the thoracic aortas, the aortas were stained with hematoxylin and eosin (H&E). The intima–media thickness (IMT) quantification demonstrated a mean of 59.5 ± 6.3 μm for the untreated ApoE mice and a strong reduction was obtained after 11 weeks of lycopene administration (52.1 ± 4.6 μm; *p* < 0.05; Figure 3A,B). Moreover, a significant reduction in the histological score was observed in the ApoE KO + HFD + Lyco group compared to the untreated animals (*p* < 0.0001 vs. ApoE KO + HFD group; Figure 3C).

### 3.5. Immunofluorescence Evaluation of Nrf2

**Figure 4:**
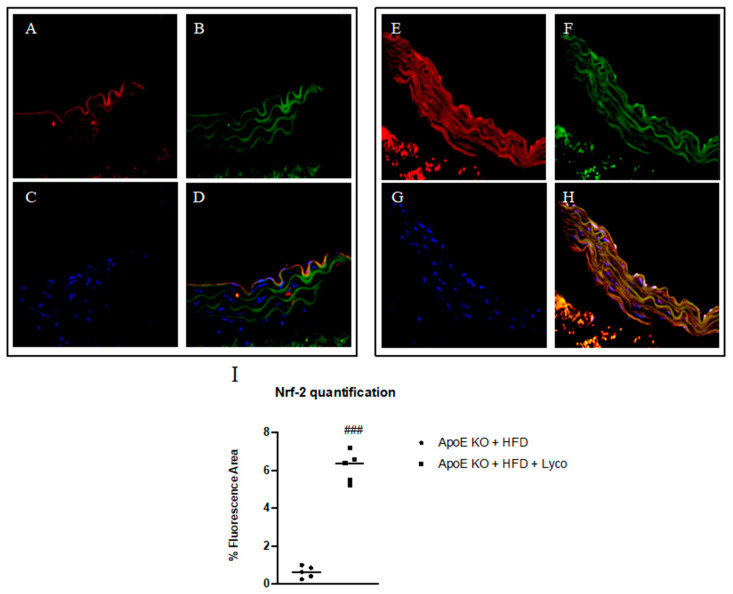
Immunofluorescence staining for Nrf2 (red channel, (**A**,**E**)) and CD34 (green channel, (**B**,**F**)) in aorta samples of ApoE KO + HFD and ApoE KO + HFD + Lycopene mice. The merge signal shows the co-localization of Nrf2 in CD34+ cells (**D**,**H**). Panels (**C**,**G**) showed nuclei stained with DAPI. The graph in (**I**) shows the quantification of Nrf2 expression performed with Image-J. Values are expressed as the mean ± SD of 5 animals. ### *p* < 0.0001 vs. ApoE KO + HFD.

**Figure 5:**
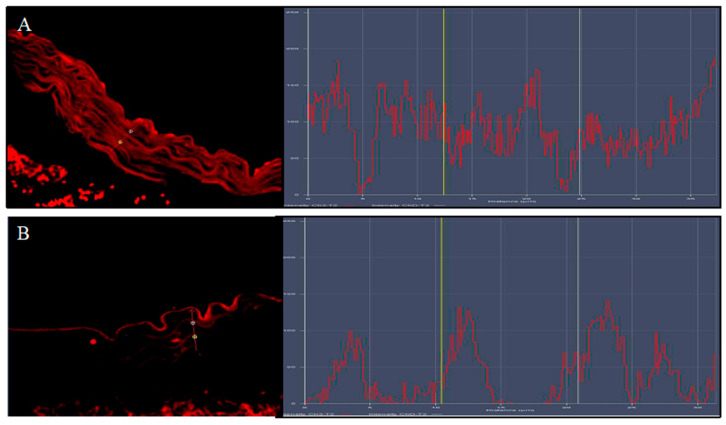
The images show the display profile obtained by the confocal laser; a high intensity of fluorescence pattern for Nfr2 was observed in ApoE KO + HFD + Lycopene mice (**A**) compared to the fluorescence pattern observed in untreated ApoE KO + HFD mice (**B**).

The thoracic aortas of the ApoE KO + HFD group exhibited the rare presence of Nrf2 positive staining (red channel, Figure 4A); the simultaneous labeling of CD34 (green channel; Figure 4B) demonstrated that Nrf2 was expressed in the endothelial CD34+ cells mainly in the tunica intima of aorta, as evidenced by the yellow fluorescence (Figure 4C). The samples taken from the ApoE KO + HFD + Lycopene group showed a significant increase in Nrf2 expression (Figure 4G) in the endothelial CD34+ cells along the tunica intima and media of the aorta compared to the untreated group, as demonstrated by the merge signal (Figure 4H). The display profile obtained by the confocal laser showed a high intensity fluorescence pattern for Nfr2 in the ApoE KO + HFD + Lycopene mice compared to the fluorescence pattern observed in the untreated ApoE KO + HFD mice (Figure 5).

### 3.6. Lycopene Effects on PPARα, SREBF-1 and AMPK-α Expression

**Figure 6:**
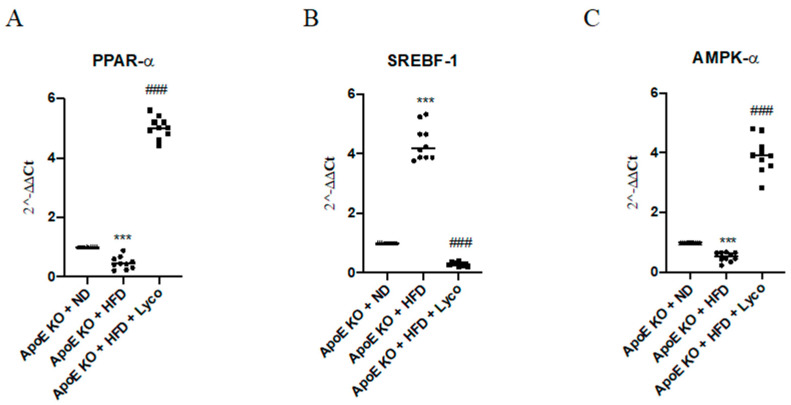
The graphs represent qPCR results of PPAR-α (**A**), SREBF-1 (**B**), AMPK-α, and (**C**) mRNA expression from liver samples. Values were obtained from 10 animals per group and are expressed as the means and SD.*** *p* < 0.0001 vs. ApoE KO + ND; ### *p* < 0.0001 vs. ApoE KO + HFD.

**Figure 7:**
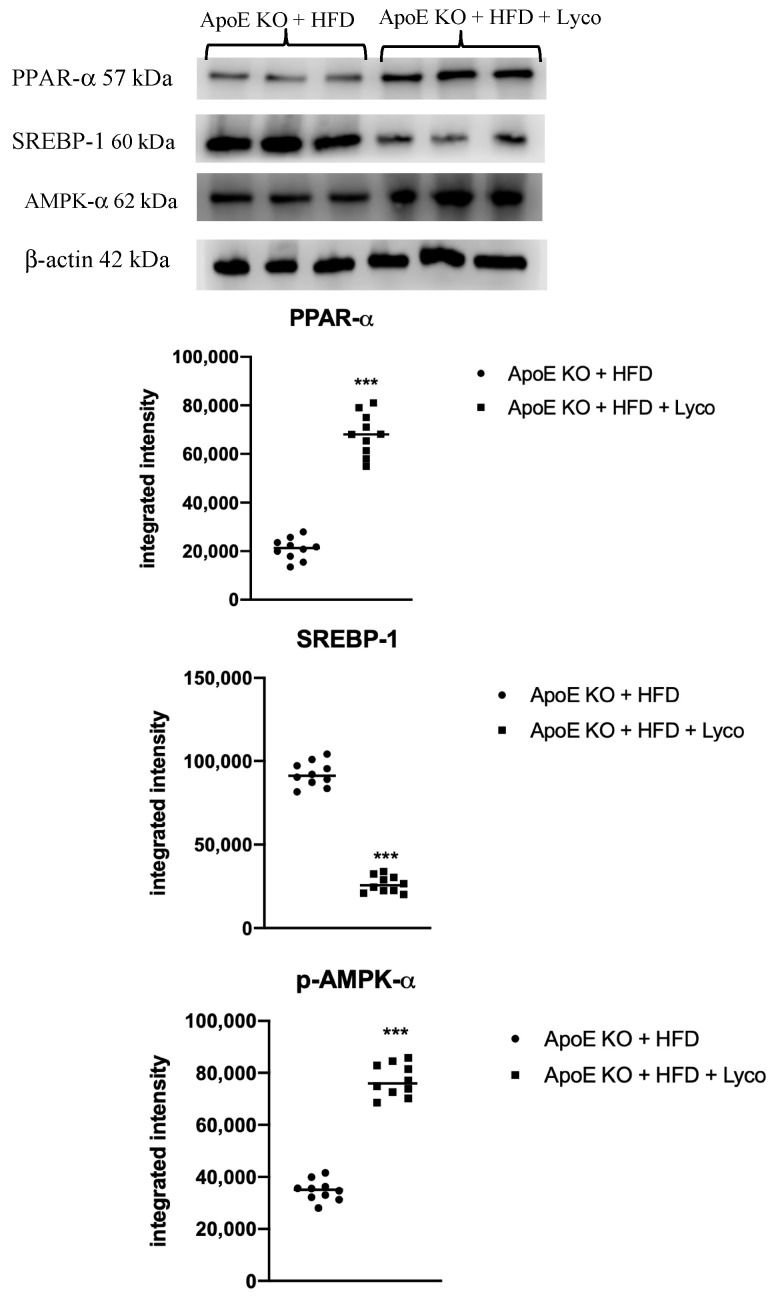
Western blot analysis of PPAR-α, SREBP-1c, and p-AMPK-α in liver samples. Values were obtained from 10 animals per group and are expressed as the means and SD. *** *p* < 0.0001 vs. ApoE KO + ND.

The animals fed with HFD showed a marked reduction of PPARα and AMPK-α mRNA expression compared to the ApoE KO + ND group (*p* < 0.0001 vs. ApoE KO + ND group; Figure 6A,C). Lycopene treatment caused a significant increase in PPARα and AMPK-α mRNA expression compared to the ApoE KO + HFD group (*p* < 0.0001 vs. ApoE KO + HFD group; Figure 6A,C). In contrast, SREBPF-1 mRNA expression was significantly increased in the ApoE KO + HFD group compared to the ApoE KO + ND group (*p* < 0.0001 vs. ApoE KO + ND group; Figure 6B) and reduced following the lycopene treatment in the HFD animals (*p* < 0.0001 vs. ApoE KO + HFD group; Figure 6B). Similar results were obtained when evaluating the protein expression of PPARα, SREBP-1, and p-AMPK-α using Western blot analysis of the liver samples (*p* < 0.0001 vs. ApoE KO + HFD group; Figure 7).

## 4. Discussion

Several mechanisms are involved in atherosclerosis development, such as the increase in the absorption of oxLDL by macrophages, the generation of Reactive Oxygen Species (ROS), and pro-inflammatory cytokines [28,29]. Lycopene demonstrated to be an effective antioxidant in several experimental models [10], and in accordance with these findings, our results showed a significant increase in Nrf2 expression, a nuclear factor responsible for encoding phase II detoxification enzymes, thus blocking phase I metabolism and metabolic activation. The increased presence of Nrf2 in the endothelium of aortas is not surprising given that prior research has shown similar results in cultured HUVEC cells through the stimulation of heme oxygenase-1 (HO-1) gene expression and the activation of the phosphoinositide 3-kinase (PI3K)/Akt pathway [30]. In addition, these results were in accordance with a previous paper that demonstrated in NZW rabbits that lycopene supplementation for 4 weeks strongly suppressed diet-induced increases in total and LDL cholesterol levels, in serum, and reduced the accumulation of cholesteryl esters in aortic tissue without affecting body weight [31]. In a longer experimental setting using the same type of animals, dietary cholesterol supplementation with lycopene reduced the intestinal absorption of cholesterol and inhibited 3-hydroxy-3-methylglutaryl coenzyme A (HMG-CoA) reductase and Acyl-coenzymeA: cholesterol acyltransferase (ACAT) activity in the liver, thus contributing to a massive reduction in plaques, despite the high cholesterol diet, and further highlighting the beneficial effects of lycopene in a well-established condition [32]. These results are suggestive of a strong antioxidant activity in the vessel wall, where atherosclerosis takes place, and thus further support the importance of this biological compound in modulating plaque development.

As plaque reduction is also a matter of decreased cholesterol and lipid accumulation, an issue that may arise involves the intake of food in the studied animals. However, no difference in body weight or food consumption has been observed throughout the study; therefore, it can be hypothesized that lycopene supplementation strongly suppressed the diet-induced increase in cholesterol and triglyceride levels at the metabolic level. Furthermore, a meta-analysis demonstrated that the consumption of 25 mg lycopene/day in humans significantly reduced total cholesterol levels, and the effects were comparable to low-dose statin treatment [33]. Additionally, a previous paper demonstrated that tomato juice supplementation could reduce triglyceride serum levels in young females that received 32.5 mg of lycopene for 8 weeks [34]. Lycopene can also modulate hepatic lipid metabolism and avoid the hepatic steatosis caused by a high-fat diet by activating SIRT1 and consequently suppressing lipogenesis, promoting lipid catabolism in the liver and skeletal muscles, and mobilizing lipids in white adipose tissue [35]. In particular, at the hepatic level, SIRT1 positively regulates the transcriptional activity of PPARα by promoting β-oxidation [18,36]; in agreement with these data, our results showed an increase in PPARα expression as a consequence of lycopene administration. The reduced lipid levels in serum may also be due to a modulation of fatty acid utilization through SREBP-1c and AMPK in the liver. In the present study, SREBP-1c expression was significantly reduced, and p-AMPK-α levels were significantly increased in the lycopene treated mice compared to the untreated group. These results were in line with previous papers that demonstrated the pivotal role of the SIRT1 agonist in reducing SREBP-1c expression and increasing AMPK activation [7,37]. Considering that the invasion of lipids into the aortic tissue is the first step in plaque progression and is required for the development of advanced aortic plaques, an increased lipid metabolism could account for a reduced accumulation of lipids within the aortic tissue with a consequent slowdown in the progression of atherosclerotic change. As a matter of fact, the analysis of atherosclerotic lesions revealed that lycopene-treated mice developed significantly fewer atherosclerotic lesions than the untreated group. Accordingly, in another study, using rabbits fed a high fat diet, lycopene showed to be similar to statins in terms of the degree of atherosclerotic lesion reduction [38], while reduced vascular inflammation and improved endothelial function have been obtained in HUVECs through the inhibition of adhesion molecule-1 (ICAM-1) expression [39]. As shown in clinical studies, lycopene has a beneficial effect on the intima–media thickness, a measure of the atherosclerosis’s severity, as high lycopene serum levels were correlated with a reduced IMT and a slow IMT progression [40,41,42] in subjects with an anti-oxidant rich diet. Our study confirmed this effect using an adjusted lycopene dose that was found effective in humans [20], demonstrating that even in a genetically biased model this flavonoid can exert beneficial effects.

Nevertheless, the present study has some limitations, for instance, we did not include animals pre-treated with lycopene as a preventive intervention nor a group fed with HFD and treated with statins as the gold-standard treatment. The preventive treatment with lycopene would have added little to the study, considering that ApoE mice represent a model of genetic atherosclerosis and develop plaques regardless of the HFD, which serves as an accelerator of the disease. Additionally, our main goal was to assess whether or not lycopene could be considered as a therapeutic option, possibly together with a proper diet or with low dose cholesterol-lowering drugs. Furthermore, additional studies should be performed to compare the efficacy of this extract to other substances with well-established antioxidant and anti-atherosclerotic activities, such as resveratrol or fish oil. Moreover, considering that atherosclerosis is related to gender [43], it could be of interest to evaluate the efficacy of lycopene treatment in both sexes, but here the results have been obtained in solely male mice. In addition, we did not assess lycopene’s anti-atherosclerotic effects in a chronic model of atherosclerosis, and such aspects should be evaluated in future studies considering a longer disease course and a human lifespan. However, in the present experiment, the efficacy of lycopene supplementation was demonstrated, indicating that lycopene may be used as a possible adjuvant treatment for the management of atherosclerosis due to its antioxidant function, lipid-lowering effects, and modulation of fatty acid utilization and oxidation. Therefore, these additional data may support the clinical use of lycopene, although this speculation needs to be proven in a clinical trial.

## Figures and Tables

**Table 1:** Primer list.

Gene	Sequence
β-actin	Fw:5′AGCCATGTACGTAGCCATCC3′
	Rw:5′CTCTCAGCTGTGGTGGTGAA3′
PPAR-α	Fw:5′AGCCCCATCTGTCCTCTCTC3′
	Rw:5′TTCGACACTCGATGTTCAGG3′
SREBF-1	Fw:5′GATCAAAGAGGAGCCAGTGC3′
	Rw:5′TAGATGGTGGCTGCTGAGTG3′
AMPK-α	Fw:5′CGAAGCTCAAGGAAAGCCCT3′
	Rw:5′CCTGGTCTTGGAGCTACGTC3′

## Data Availability

The data presented in this study are available on request from the corresponding author.
